# Sleep Duration Affects Appetite-Regulating Hormones

**DOI:** 10.1371/journal.pmed.0010068

**Published:** 2004-12-07

Some of us, when awake in the middle of the night, feel an urge to visit the kitchen. This could explain results of previous studies that have shown a link between short sleep duration and high body mass index (BMI). But a study by Emmanuel Mignot and colleagues suggests that it's not just the additional snacking opportunities that make short sleepers more likely to be overweight.

Intrigued by the connection between sleep and BMI, and by recent studies showing that sleep deprivation in laboratory settings can cause a decrease in serum levels of leptin, a hormone known to control appetite, Emmanuel Mignot and colleagues set out to study the levels of various hormones known to regulate appetite and energy expenditure under “real life” conditions.

They took advantage of the Wisconsin Sleep Cohort Study, an ongoing longitudinal study of sleep habits and disorders in the general population. The study began in 1989, when researchers mailed state employees aged 30–60 years a survey on sleep habits, health, and demographics. Mail surveys were repeated at 5-year intervals, and some of the respondents were recruited to sleep a night in the laboratory and undergo various tests. A number of participants were also asked to keep a sleep diary for 6 days. The study has already shown connections between sleep apnea and hypertension, and between menopause and sleep-disordered breathing.

For their study, Mignot and colleagues measured sleep duration (habitual and immediately prior to blood sampling), BMI, and pre-breakfast blood hormone levels in 1,024 participants. Consistent with previous studies, they found that in individuals who sleep less than 8 hours (74% of all participants), BMI was inversely proportional to sleep duration. In addition, short sleep was associated with low leptin and high ghrelin levels (ghrelin is a hormone thought to stimulate food intake).These hormonal differences are likely to increase appetite, which could be responsible for the increased BMI in short sleepers.[Fig pmed-0010068-g001]

**Figure pmed-0010068-g001:**
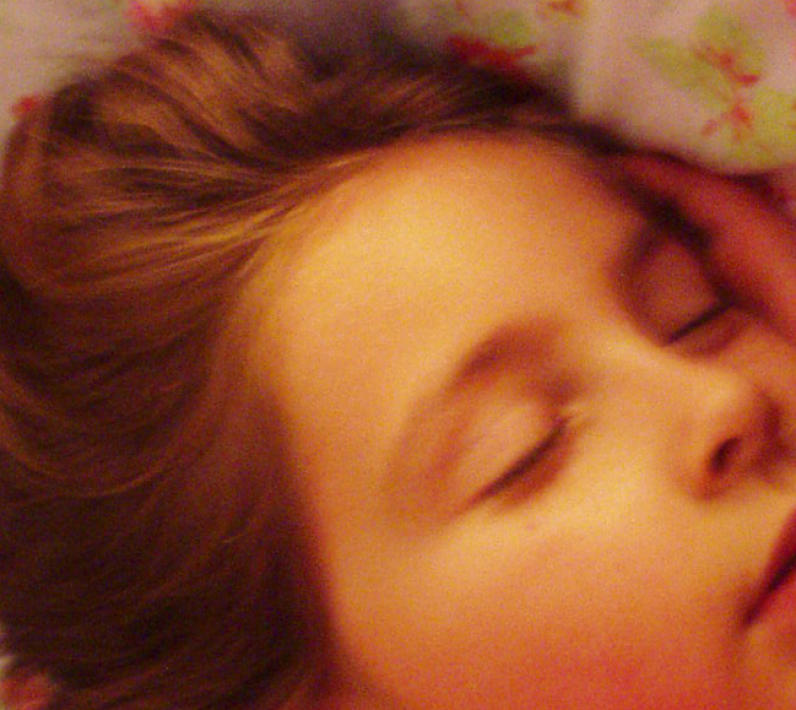
Peaceful sleep (Photo: Sharad Taheri)

These findings could explain, at least in part, why societies in which excess calories are much easier to come by than a good night's sleep are more prone to obesity. Mignot and colleagues plan to test this in intervention studies where they make people sleep more and measure the effects on body mass. “Good sleep, healthy eating habits, and regular exercise each may have important roles in fighting obesity in modern society,” suggests Mignot.

